# Direct-Acting Antivirals for the Treatment of Chronic Hepatitis C: Open Issues and Future Perspectives

**DOI:** 10.1155/2013/704912

**Published:** 2013-06-05

**Authors:** Hee Bok Chae, Seon Mee Park, Sei Jin Youn

**Affiliations:** Department of Internal Medicine, Chungbuk National University Hospital, Chungbuk National University College of Medicine, 1 Sunwhan-ro, Heungdok-gu, Cheongju 361-711, Republic of Korea

## Abstract

Currently, two direct-acting antivirals (DAAs) show well-established efficacy against hepatitis C virus (HCV), namely, first-wave protease inhibitors telaprevir and boceprevir. Most clinical trials have examined DAAs in combination with standard of care (SOC) regimens. Future therapeutic drugs were divided into three categories. They are second-wave protease inhibitors, second-generation protease inhibitors, and polymerase inhibitors. Second-wave protease inhibitors are more improved form and can be administered once a day. Oral drug combinations can be favored because interferon (IFN) not only has to be given as intradermal injection, but also can cause several serious side effects. Combination of drugs with different mechanisms shows a good sustained virological response (SVR). But several mutations are associated with viral resistance to DAAs. Therefore, genotypic resistance data may provide insights into strategies aimed at maximizing SVR rates and minimizing resistance. Combined drug regimens are necessary to prevent the emergence of drug-resistant HCV. Many promising DAA candidates have been identified. Of these, a triple regimen containing sofosbuvir shows promise, and treatment with daclatasvir plus asunaprevir yields a high SVR rate (95%). Oral drug combinations will be standard of care in the near future.

## 1. Introduction

Until now, combined treatment with pegylated interferon-*α* (PegIFN-*α*) and ribavirin (RBV) (known as PR therapy) has been the standard of care (SOC) for patients chronically infected with hepatitis C virus (HCV). However, direct-acting antiviral agents (DAAs) are assuming a more prominent role. At present, only two first-generation DAAs (telaprevir (TVR) and boceprevir (BOC)) are available, although many other candidate DAAs are being developed. TVR and BOC are used only in developed countries to treat patients chronically infected with HCV. They are not used commonly in developing countries because of their high cost. 

**Figure 1:**
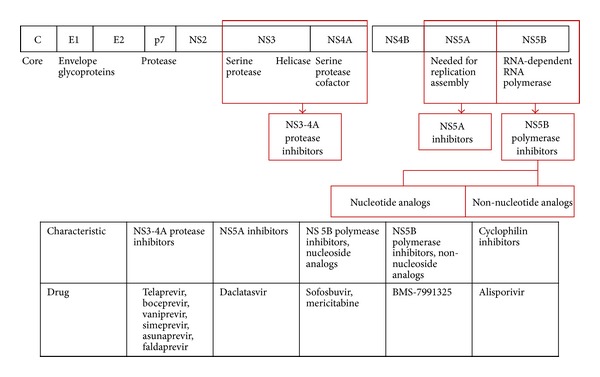
Targets for direct-acting antivirals. Modified from [2].

We can classify DAAs according to their action sites, such as protease inhibitor, polymerase inhibitor, NS5B inhibitor, and NS5A inhibitor. The main mechanism of action of DAAs is the inhibition of enzyme, for example, protease or polymerase, but the NS5A inhibitor has a different mechanism of action from other DAAs. It inhibits the assembly of this replication complex (Table 1) (Figure 1) [1, 2]. Another approach to HCV therapy is to target the host factors that the virus uses for its own life cycle, for example, cyclophilin inhibitors or nitazoxanide. In this paper, we will focus on only DAAs and will not cover other treatment options, like cyclophilin, HCV vaccine. We will discuss the efficacy and limitations of both currently approved and new candidate drugs. 

## 2. Currently Available DAAs

In May 2011, the U.S. Food and Drug Administration (FDA) approved TVR and BOC for use in combination therapies with PegIFN-*α* and RBV for adult patients chronically infected with HCV genotype 1. The drugs are used to treat patients with compensated liver cirrhosis, who are treatment-naïve or who have been previously treated with IFN-based regimens [3, 4]. Both TVR and BOC inhibit the viral NS3/4A serine protease, which is essential for replication [5, 6].

### 2.1. Telaprevir (TVR)

Three phase III clinical trials have been conducted to evaluate the efficacy of TVR when administered to treatment-naïve chronic HCV (genotype 1) patients in combination with PegIFN-*α*-2a and RBV [7, 8]. In the ADVANCE trial, patients received TVR together with PegIFN-*α* and RBV (PR) for either 8 (T8PR) or 12 (T12PR) weeks, followed by PegIFN-*α* or RBV (PR) alone in a response-guided therapy [7]. Extended rapid virological response (eRVR) was defined as undetectable HCV RNA levels at weeks 4 and 12. The patients who did not achieve an eRVR received PegIFN-*α* plus RBV for a total of 48 weeks. The overall SVR rates for patients in the T8PR and T12PR groups were 69% and 75%, respectively. The SVR rate for the control group with only PR was 44% [7].

The ILLUMINATE, another TVR trial, focused on defining the utility of response-guided therapy (RGT) in patients that did achieve an eRVR. All patients received an initial 12-week course of TVR-based triple therapy, followed by treatment with PegIFN-*α* plus RBV [8]. Patients who achieved an eRVR at week 20 were randomized to receive either an additional 3- or 28-week course of PegIFN-*α* plus RBV. The overall SVR rate for all patients was 72%. The SVR rates for those patients (65%) who achieved an eRVR and received either an additional 3- or 28-week course of PegIFN-*α* plus RBV were 92% and 88%, respectively.

The REALIZE, the third trial of TVR, was conducted for patients who experienced treatment failure after SOC therapy [9]. The clinical trial had three arms. Patients in the first arm received T12PR triple therapy for 12 weeks, followed by a placebo plus PR for 4 weeks and then PR alone for 32 weeks. The patients in the second arm received placebo plus PR (lead-in phase) for the first 4 weeks, followed by TVR-based triple therapy for 12 weeks and then PR alone for 32 weeks (48 weeks in total). The patients in the third arm received PR alone for 48 weeks (control group). The overall SVR rates for the three groups were 64%, 66%, and 17%, respectively. The best response rate was observed for those patients in each group that had previously relapsed after PR therapy (83%, 88%, and 24%, resp.) [9]. 

In summary, the triple regimen including TVR showed good response in genotype 1 patients. The SVR rate can be maximized using a response-guided paradigm. The triple regimen was also effective in treatment-failure patients, especially who relapsed after PR therapy. 

### 2.2. Boceprevir

**Figure 2:**
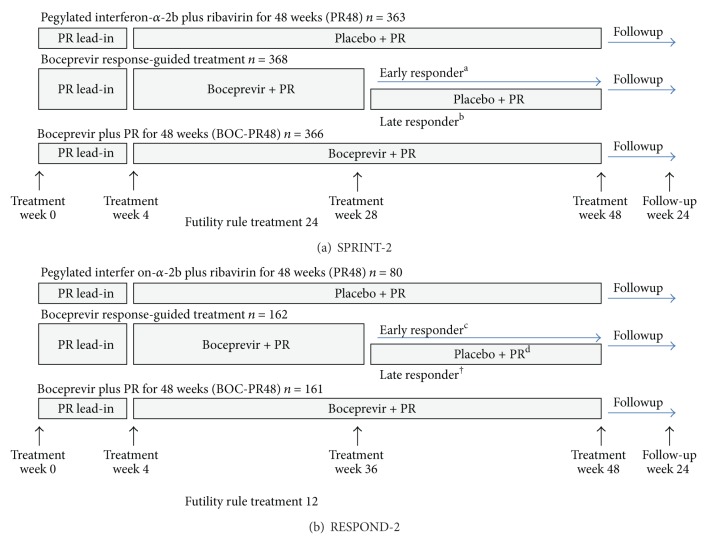
Phase III trials of boceprevir in patients with hepatitis C genotype-1 infection. (a) SPRINT-2 trial in previously untreated patients. (b) RESPOND-2 trial for previously treated patients; patients were partial responders and relapsers and null-responders. PR: pegylated interferon-*α*-2b 1.5 *μ*g/kg per week plus weight-based ribavirin 600–1400 mg per day. BOC: boceprevir 800 mg every 8 h. ^a^Hepatitis C RNA treatment weeks 8–24 undetectable. ^b^Hepatitis C RNA treatment week 8 detectable, treatment week 24 undetectable. ^c^Hepatitis C RNA treatment weeks 8–12 undetectable. ^d^Hepatitis C RNA treatment week 8 detectable, treatment week 12 undetectable. Excerpted from Pearlman [11].

Let us look at two important phase III clinical trials on BOC. The first one, SPRINT-2, evaluated the efficacy of BOC in two cohorts of treatment-naïve patients [10]. All patients were first treated with a lead-in therapy comprising PegIFN-*α*-2b plus weight-based RBV for a period of 4 weeks, followed by one of three regimens. After the lead-in, patients were assigned to one of three groups. (1) Group 1, PegIFN-*α*-2b, RBV, and placebo for an additional 44 weeks. (2) Group 2, BOC, PegIFN-*α*-2b, and RBV for an additional 24 weeks, followed by 20 more weeks of PegIFN-*α*-2b if HCV RNA was detectable at weeks 8 and 24. (3) Group 3, BOC, PegIFN-*α*-2b, and RBV for an additional 44 weeks, that is, SOC therapy (Figure 2) [11]. The overall SVR rates were higher in the BOC-treated arms (63% and 66%) than in the SOC arm (38%), but differed according to race. In black patients, the SVR rates were 42% in the RGT arm, 53% in the fixed duration arm, and 23% in the SOC arm.

The RESPOND-2 trial was a phase III clinical trial [12]. The subjects were prior partial responders or relapsers with PegIFN-*α*-2b and RBV. Null responders were not studied in this trial. (1) Group 1, PegIFN-*α*-2b, RBV, and placebo for an additional 44 weeks. (2) Group 2, BOC, PegIFN-*α*-2b, and RBV for an additional 32 weeks, followed by 12 more weeks of PegIFN-*α*-2b and RBV if HCV RNA was detectable at week 8, but undetectable at week 12. (3) Group 3, BOC, PegIFN-*α*-2b, and RBV for an additional 44 weeks. Therapy was discontinued in patients who were HCV RNA positive at week 12 (Figure 2) [11]. The overall SVR rates at week 24 were 21%, 59%, and 66%, respectively in Group 1, Group 2 (RGT), and Group 3 (48 weeks). These triple therapy appear to yield even higher rates of SVR, 29, 69, and 75% in prior relapsers than in partial responders (7%, 40%, and 50%).

### 2.3. Vaniprevir (MK-7009)

Vaniprevir is a macrocyclic hepatitis C virus nonstructural protein 3/4A protease inhibitor. Treatment-naïve patients with HCV genotype 1 infection were randomized to receive open-label PegIFN and RBV in combination with blinded placebo or vaniprevir (300 mg bid, 600 mg bid, 600 mg qd, and 800 mg qd) for 28 days, and then open-label PegIFN and RBV for an additional 44 weeks. Across all doses, vaniprevir was associated with HCV RNA levels approximately 3 log_10_ IU/mL lower in vaniprevir-treated patients, compared to placebo recipients. Rates of RVR were significantly higher in each of the vaniprevir dose groups, compared to the control regimen (68.8%–83.3% versus 5.6%; *P* < 0.001 for all comparisons). Vomiting appeared to be more common at higher vaniprevir doses (40% in 600 mg bid group) [13].

### 2.4. Preliminary Data from Patients with Other Genotypes Treated with DAAs

#### 2.4.1. HCV Genotype 2

The SVR rate for patients infected with HCV genotype 2 and treated with SOC is almost 80%. There is no space for DAAs to show any increase of treatment effect because it is enough high. DAAs may be less effective in this patient group than in patients infected with HCV genotype 1. TVR, the first agent to directly target viral replication, is effective against HCV-2 but not against HCV-3 (see below). Foster et al. evaluated combined treatment with TVR plus PegIFN-*α*-2a and RBV in five patients infected with HCV-2 and compared the results with those obtained after treating nine patients with TVR alone or treating nine patients with PR (control group). Triple combination therapy yielded an SVR rate of 100%, which is remarkable considering the 89% rate observed in patients receiving standard PR [26]. Other NS3/4A protease inhibitors, nucleoside and non-nucleoside reverse replicase inhibitors, and NS5A inhibitors have antiviral activity against HCV-2. One of the most promising drugs is a nucleotide analogue polymerase inhibitor called PSI-7977 [27]. An open-label study (the PROTON study) evaluated the efficacy of PSI-7977 in 15 patients infected with HCV-2, in 10 patients infected with HCV-3, and in a larger group of patients with HCV-1 infection [28]. That study reported an RVR of 96% after the triple combination of 400 mg of PSI-7977 plus PR. Twenty-four HCV-2 and HCV-3 patients who completed the 12 weeks of treatment achieved SVR (96%).

#### 2.4.2. HCV Genotype 3

The SVR rate for patients infected with HCV genotype 3 and treated with SOC is almost 80% [29]. TVR and BOC have revolutionized the treatment of genotype 1 HCV. Indeed, both have recently been recommended for use in combination with standard PR regimen for the treatment of patients chronically infected with HCV genotype 1. However, both BOC and TVR are ineffective against HCV-3. 

### 2.5. Future Therapeutic Options

Two available DAAs, TVR and BOC, have several limitations. The role of these drugs is a supplement to PegIFN. These two drugs can cause severe side effects, for example, anemia, rash, and hyperbilirubinemia. Lastly, their dosing schedule is three times a day. The therapeutic drugs that are being developed for future use try to resolve these limitations of currently available DAAs. These new drugs fall into three categories: second-wave protease inhibitors, second-generation protease inhibitors, and polymerase inhibitors. 

#### 2.5.1. Second-Wave Protease Inhibitors

Second-wave protease inhibitors offer several advantages over currently available drugs. In the near future, improved pharmacokinetics will allow a once-a-day dosing schedule, which means that the side-effect profiles should be more tolerable. Second-wave protease inhibitors have similar genotype coverage and similar resistance profiles to those of TVR and BOC and will replace the first-generation protease inhibitors currently used for PR combination therapy, thereby becoming the initial partners in the first generation of “all-oral regimens.” 

Simeprevir (TMC435) is an NS3/4A protease inhibitor that is taken orally once per day; the drug is currently undergoing phase III clinical trials for the treatment of HCV infection [30]. The PILLAR study (a phase IIb trial) was designed to test the efficacy of simeprevir when used in combination with PR for either 24 or 48 weeks. An SVR was achieved in 68–76% of patients treated with this triple therapy regimen, and approximately 80% of subjects were eligible to receive shortened 24 weeks of therapy. The result of subgroup analysis was very high SVR (93–96%) [14]. Adverse effects were similar to those observed after SOC therapy. The lowest rate of relapse (8%) was found in the study arm receiving TMC 435 (150 mg/day) plus PR for 24 weeks. 

The ASPIRE trial was a phase IIb trial for genotype 1 patients who had failed previous treatment with PR therapy. All patients received PR for 48 weeks. The best results were observed in the group treated with triple therapy with simeprevir 150 mg (SVR) plus PR in comparison with placebo plus PR, which achieved an SVR of 85% versus 37% in prior relapsers, 75% versus 9% in partial responders, and 51% versus 19% in prior nonresponders [15].

Faldaprevir (BI201335) is another NS3/4A protease inhibitor that has completed phase II testing (the SILEN-C1 study) and can be administered using a once-per-day dosing schedule. The treatment regimen included BI201335 in addition to PR for 24 weeks at doses of 120 and 240 mg, followed by another 24 weeks of standard therapy [17]. The overall SVR rate was 83% for the 240 mg dose. Ninety-two percent of the patients that showed an eRVR also achieved an SVR, regardless of the duration of subsequent PR therapy. Adverse events (mostly gastrointestinal) meant that treatment was discontinued in 7.3% of subjects. 

Asunaprevir (BMS-650032) is a twice-daily protease inhibitor that is being developed for use with daclatasvir (an NS5A inhibitor) and BMS 791325 (a non-nucleoside inhibitor) in both IFN-containing and IFN-free regimens. Asunaprevir plus daclatasvir was the first regimen to cure HCV-infected patients without the need for IFN [16]. However, asunaprevir is not an ideal protease inhibitor because a twice-per-day schedule may be associated with hepatotoxicity. 

#### 2.5.2. Second-Generation Protease Inhibitors

Two second-generation protease inhibitors, MK-5172 and ACH-2684, are currently under clinical trial. 

MK-5172 is a novel macrocyclic NS3/4a protease inhibitor that is currently undergoing phase II clinical trials. R155 is the main overlapping position for drug resistance, and different mutations at this site within the NS3 protease confer resistance to nearly all protease inhibitors. However, MK-5172 shows potent antiviral activity against HCV viruses harboring mutations at position R155. Based on its preclinical profile, MK-5172 is expected to have broad-spectrum activity against multiple HCV genotypes (including genotype 3) and other clinically important drug-resistant variants. Indeed, trials in genotype-1-positive patients show that 75% had HCV RNA levels below the limit of detection. In addition, the drug was generally well tolerated [31].

ACH-2684 is a macrocyclic, noncovalent, reversible inhibitor of the NS3 protease. Phase Ib clinical trials showed that administration of ACH-2684 to patients infected with HCV genotype 1 achieved a mean 3.73 log_10_ reduction in HCV RNA levels after 3 days of monotherapy at a single dose of 400 mg/day. In addition, ACH-2684 was safe and well tolerated [32]. Thus, this drug shows great promise, although further clinical trials are needed. 

#### 2.5.3. Polymerase Inhibitor-Nucleoside Inhibitors

Two HCV nucleos(t)ide analogues have entered phase II/III clinical trials: mericitabine and sofosbuvir.

#### 2.5.4. Nucleoside Inhibitors in Clinical Trials with Interferon

Mericitabine (RG 7128): the JUMP-C trial (phase II) investigated the safety and efficacy of mericitabine (RG 7128) (1000 mg bid) plus PR after 24 weeks of response-guided therapy. The overall SVR rates were higher in patients treated with mericitabine plus PR than in patients treated with PR alone (58% versus 36%) [22].

Sofosbuvir (GS-7977): the ATOMIC study (another phase II trial) evaluated combined treatment with sofosbuvir plus PR in 316 noncirrhotic patients infected with HCV genotypes 1, 4, or 6. This study evaluated the proper duration of treatment for genotype 1 patient. Patients infected with HCV genotype 1 were randomized into two groups: one group received sofosbuvir plus PegIFN/RBV for 12 or 24 weeks, and the other received sofosbuvir plus PR for 12 or 24 weeks, followed by rerandomization (1 : 1) into two further groups that received either an additional 12 weeks of sofosbuvir alone or an additional 12 weeks of sofosbuvir plus RBV. The results of an interim analysis showed that patients who received 12 weeks of therapy with the triple combination of sofosbuvir plus PR achieved SVR rates of 90% [20].

#### 2.5.5. Nucleoside Inhibitors in Clinical Trials without Interferon

Mericitabine (RG 7128): the INFORM-1 study provided the first proof of principle that combined treatment with mericitabine plus danoprevir (an NS3/4 protease inhibitor) in the absence of IFN is effective at reducing HCV RNA levels. At day 14, the highest combined dose (1000 mg mericitabine and 900 mg danoprevir bid) resulted in a median −5.1 log_10_ IU/mL reduction in HCV RNA levels in treatment-naïve patients and a median −4.9 log_10_ IU/mL reduction in HCV RNA levels in patients that did not respond to previous PR therapy [23].

The INFORM-SVR trial (a phase IIb trial) evaluated the efficacy of a 12- or 24-week interferon-free regimen comprising ritonavir-boosted danoprevir (DNV/r, 100 mg/100 mg) plus mericitabine (1000 mg, bid), either with or without RBV, in treatment-naïve patients infected with HCV genotype 1. The data showed that 71% of the patients infected with HCV genotype 1b who received 24 weeks of DNV/r, mericitabine, and RBV achieved an SVR; however, only 26% of patients infected with genotype 1a achieved an SVR. Higher SVR rates were reported in patients who were rapid virological responders [24]. 

Sofosbuvir (GS-7977/PSI-7977): the ELECTRON trial evaluated the efficacy of sofosbuvir plus RBV in the absence of IFN. The results showed that treatment-naïve patients infected with HCV genotypes 2 or 3 achieved an SVR rate of 100%. In addition, patients infected with HCV genotype 1, who did not respond to previous treatment with PR, received sofosbuvir plus RBV for 12 weeks; however, 89% of patients relapsed after the end of treatment [21]. 

#### 2.5.6. Interferon-Free Combination Trials

The SOUND-C2 study (faldaprevir plus BI 207127, with or without RBV): the Sound-C2 study is an open-label, randomized, phase IIb study of 362 treatment-naïve patients infected with HCV genotype 1 who were allocated to one of five treatment arms [18]. The final results showed that up to 85% of HCV patients infected with genotype 1b achieved an SVR. The optimal regimen was 28 weeks of faldaprevir (120 mg once daily), and BI 207127 (600 mg bid). The overall SVR rate was 70%, compared with 85% in the prevalent genotype-1b patient subgroup [19].

The Aviator study (ABT-450/r, ABT-267, or ABT-333 plus RBV): the Aviator phase IIb study assessed the safety and efficacy of ABT-450/r, ABT-267, or ABT-333 plus RBV (administered for 8, 12, or 24 weeks) in noncirrhotic treatment-naïve patients and in patients who did not respond to previous treatment with PR [33]. The SVR in treatment-naïve patients infected with genotype 1 HCV was 97.5% after 12 weeks, whereas the SVR in PR nonresponders infected with genotype 1 was 93.3%. Treatment-naïve patients infected with genotype 1a achieved an SVR of 96% after 12 weeks, whereas PR nonresponders achieved an SVR of 89%. For those patients infected with genotype 1b, the SVR was 100% for both treatment-naïve and PR nonresponders. 

Daclatasvir plus sofosbuvir with or without RBV: this trial was designed to test the efficacy of combined treatment with daclatasvir plus sofosbuvir against HCV genotypes 1, 2, and 3. Daclatasvir plus sofosbuvir were administered, either with or without RBV, for 12 or 24 weeks and either with or without a 7-day run-in with sofosbuvir [34]. A total of 44 patients infected with genotypes 2 or 3 HCV were enrolled in three arms: one arm comprised a 7-day run-in with sofosbuvir followed by 23 weeks of daclatasvir plus sofosbuvir; another arm comprised daclatasvir plus sofosbuvir for 24 weeks; and the other comprised daclatasvir plus sofosbuvir plus RBV for 24 weeks. Eighty-eight percent of patients in the first group achieved an SVR at week 12, compared with 100% in the second group and 86% in the third group.

Daclatasvir, asunaprevir, and BMS-791325: daclatasvir is the first NS5A replication complex inhibitor to be investigated in HCV clinical trials and is currently in phase III of development. Asunaprevir is an NS3 protease inhibitor that is also undergoing phase III development along with daclatasvir. BMS-791325 is a non-nucleoside inhibitor of the NS5B polymerase and is currently undergoing phase II development as a component of daclatasvir-based treatment regimens. This phase II study examined the efficacy of these DAAs in HCV G1 treatment-naïve patients [25]. The trial split patients into two groups. Group 1 received a 24-week course of daclatasvir, asunaprevir, and BMS-79132. Group 2 received a 12-week course of daclatasvir, asunaprevir, and BMS-79132. The result was that 94% of patients showed an undetectable viral load at week 4 and at the end of the trial in Group 1. One hundred percent of patients had an undetectable viral load at the end of the trial in group 2. 

### 2.6. Optimized Treatment Algorithms for the Management of HCV Patients

**Figure 3:**
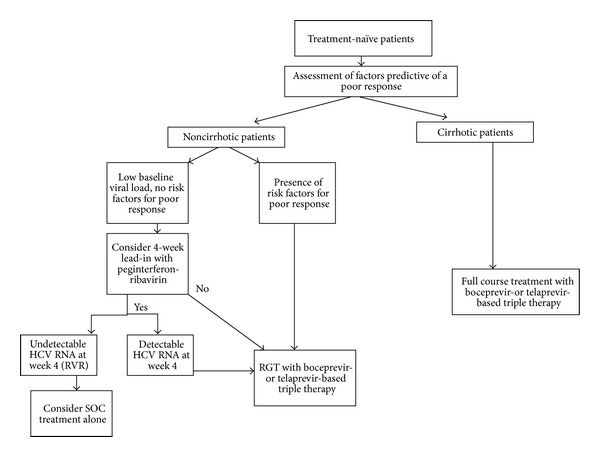
Proposed algorithm for the use of protease inhibitors in treatment-naïve HCV genotype 1 infected patients. Pretreatment assessment should include careful consideration of lifestyle factors, comorbid conditions, potential drug interactions, and assessment for the presence of cirrhosis. In noncirrhotic patients, the presence of factors predictive of a poor response to therapy should be patients with no risk factors for a poor response to therapy; the decision to use a 4-week lead-in with peginterferon and ribavirin and to continue on SOC in those who achieve an RVR should only be taken following careful and balanced discussion with the patient. Excerpted from Ramachandrean et al. [35].

**Figure 4:**
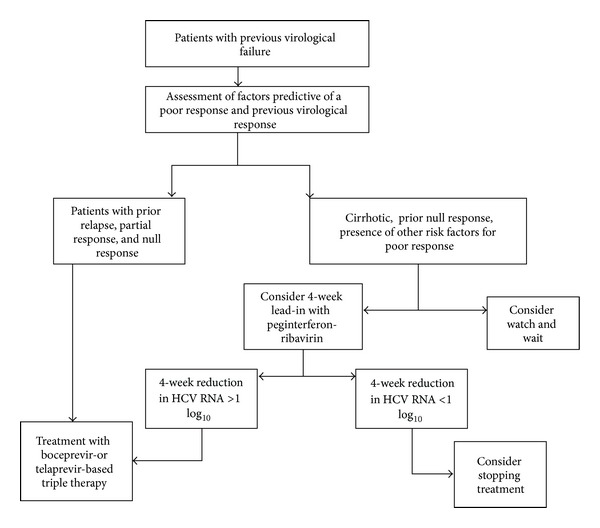
Proposed algorithm for the use of protease inhibitors in HCV genotype 1 infected patients who have had previous virological failure on treatment. Pretreatment assessment should include careful consideration of lifestyle factors, comorbid conditions, potential drug interactions, assessment for the presence of cirrhosis, and the presence of factors predictive of a poor response to therapy. Identification of the degree of previous response should be attempted. If this information is not available, patients should be considered as prior null responders to maximize cure rates. In cirrhotic prior null responders, the decision to watch and wait for novel therapies or to use a 4-week lead-in with peginterferon and ribavirin to identify patients more likely to achieve an SVR should only be taken following careful and balanced discussion with the patient. Excerpted from Ramachandrean et al. [35].

This paper did not focus on general approaches for treating patients that are chronically infected with HCV. Instead, it focused on treatments based on DAAs and particularly on clinical trials of DAAs that target HCV genotype 1. HCV genotypes 2 and 3 can be effectively treated with current SOC therapy. Genotype 4 is the most difficult genotype to treat. The standard treatment for HCV genotype 4 is a 48-week course of PR. Furthermore, patients infected with HCV genotype 4 who have previously relapsed, or are non-responders, are unlikely be cured by the PR regimen. The optimized treatment algorithms are shown in Figures 3 and 4 [35].

## 3. Conclusion

In conclusion, only two DAAs have been approved for the treatment of patients infected with HCV (TVR and BOC). Both are used in combination with PR therapy. Although several clinical trials examined the efficacy of IFN-free regimens (to avoid the side effects associated with IFN), most clinical trials have examined the efficacy of DAAs when used in combination with IFN. Response-guided therapy using the PegIFN-*α* regimen can be used with DAA therapy to select nonresponders. TVR and BOC play an important role in the treatment of patients chronically infected with HCV genotype 1. Genotypes 2 and 3 (but not genotype 4) can be effectively treated with SOC therapy. Of the emerging second-generation treatments, a triple regimen containing sofosbuvir shows great promise in terms of treatment efficacy. In addition, the combination of two oral drugs (daclatasvir and asunaprevir) achieved a high SVR rate (95%). Another oral drug combination (mericitabine and danoprevir) was examined in the INFORM study and achieved an SVR rate of 71%. Thus, future regimens may not require the use of IFN injections. Drug resistance will become a problem in the field of chronic HCV research; however, current data suggest that it is not yet a significant factor. 

## Figures and Tables

**Table 1:** Characteristics of HCV direct-acting antiviral classes.

Characteristic	Protease inhibitors	Protease inhibitors	Polymerase inhibitors	Polymerase inhibitors	NS5A inhibitors
	First generation	Second generation	Nucleoside analogs	Non-nucleoside analogs	

Potency	High Variable among HCV genotypes	High Variable	Moderate Consistent across genotypes	Variable Variable among HCV genotypes	High Multiple HCV genotypes

Barrier to resistance	Low	Low	High	Very low	Low

PK	Variable qd-tid	qd	qd	Variable qd-tid	qd

Adverse event	Rash (SJS, TEN), anemia, hyperbilirubinemia appetite loss, renal toxicity, elevation of uric acid	Anemia hyperbilirubinemia	Mitochondrial nuclear interaction (RBV)	Variable	Variable

Drug	Telaprevir Boceprevir	Simeprevir Asunaprevir Faldaprevir	Sofosbuvir Mericitabine	BMS-791325	Daclatasvir

Clinical trial	TVR: ADVANCE [7], ILLUMINATE [8], REALIZE [9] BCV: SPRINT-2 [10], RESPOND-2 [12]	SMV: PILLAR [14], ASPIRE [15] ASV: AI447-011 [16] FDV: SILEN-C2 [17], SOUND-C2 [18, 19]	SOF: ATOMIC [20], ELECTRON [21] MRB: JUMP-C [22], INFORM-1 [23], INFORM-SVR [24]		DCT: AI447-011 [25]

Comments		Better barrier, pan-genotypic	Single target active site	Allosteric, many targets	Multiple antiviral mechanisms of action

PK: pharmacokinetics; qd: once a day; tid: three times a day; RBV: ribavirin.

Modified from [1].

SJS: Stevens-Johnson syndrome; TEN: toxic epidermal necrolysis; TVR: telaprevir; BCV: boceprevir; SMV: simeprevir; ASV: asunaprevir; FDV: faldaprevir; SOF: sofosbuvir; MRB: mericitabine; DCV: daclatasvir.
